# Role of motor function and lung function in pathways to ageing and decline

**DOI:** 10.1007/s40520-020-01494-3

**Published:** 2020-02-13

**Authors:** Deborah Finkel, Marie Ernsth Bravell, Nancy L. Pedersen

**Affiliations:** 1 Department of Psychology, Indiana University Southeast, New Albany, IN USA; 2 School of Health and Welfare, Jönköping University, Jönköping, Sweden; 3 Department of Medical Epidemiology and Biostatistics, Karolinska Institute, Stockholm, Sweden; 4 Department of Psychology, University of Southern California, Los Angeles, CA USA

**Keywords:** Lung function, Motor function, Physical function, Longitudinal, Ageing, Disablement

## Abstract

**Background:**

Extensive research has investigated the association between age changes in various domains, including lung function and motor function. However, a few analyses have tested models that incorporate bidirectional longitudinal influences between lung and motor function to test the temporal chain of events in the disability process. Dual change score models (DCSM) assist with identification of leading indicators of change by leveraging longitudinal data to examine the extent to which changes in one variable influence subsequent changes in a second variable, and vice versa.

**Aims:**

The purpose of the current-analysis study was to apply DCSM to data from the Swedish Adoption/Twin Study of ageing to examine the nature of the longitudinal relationship between motor functioning and lung function.

**Methods:**

Three motor functioning factors were created from 20 performance measures, including measures of balance, flexibility, and fine motor skills. Peak expiratory flow measured lung function. Participants were 829 adults aged 50–88 at the first of 9 waves of testing covering a 27-year follow-up period; 80% participated in at least three waves.

**Results:**

Model comparisons indicated that decline in lung function preceded and contributed to subsequent decline in motor function.

**Discussion:**

Combined with previous results, these results suggest that declining lung function results in increasing difficulties in motor function, which contribute to subsequent declines in multiple domains.

**Conclusion:**

Understanding the cascade of events that can lead to dependence can help in the development of interventions targeted early in the disablement process.

**Electronic supplementary material:**

The online version of this article (10.1007/s40520-020-01494-3) contains supplementary material, which is available to authorized users.

## Introduction

Documented increases in lifespan can translate to an intensified challenge to minimize years lived with morbidity and dependence, and to maximize quality of the additional years of life [1, 2]. Physical functioning and cognitive functioning are essential to the maintenance of independence in older individuals, particularly the ability to remain mobile [3]. Mobility measures such as balance and gait have been shown to predict the onset of difficulties with activities of daily living that are foundational to independent living [4, 5]. Identification of the causal chain that leads from independence to dependence may enable health practitioners to identify older adults at risk for dependence and to implement appropriate interventions at the optimal point in the progression.

Models of the disablement process posit multiple factors that lead to eventual disability, highlighting a pathway from impairments of function (musculoskeletal and cardiovascular) to functional limitations (walking and balancing) to disability [6]. Extensive research has focused on the relationship between physical function and functional limitations, including investigations of the association between lung function and motor function. Impaired lung function can reduce the energy supply available for mobility, resulting in greater fatigue and increased time required to perform daily tasks, even if the tasks can be completed without assistance [7, 8]. Research indicates that reductions in balance and mobility are found in patients with challenges to lung function, such as chronic obstructive pulmonary disease [9]. Even in healthy older adults, lung function and respiratory muscle strength were significantly correlated with walking speed and other measures of physical performance in cross-sectional studies [3, 10, 11].

Stronger tests of the temporal chain of events in the disablement process require longitudinal data. In an early study of Swedish adults aged 84–90 years at intake, measured lung function (peak expiratory flow rate) predicted stability in self-reported mobility over 2 years, but not over 4 years of follow-up [12]. In a sample of Danish adults interviewed at ages 75 and 80, better lung function and walking speed both contributed to prediction of self-reported mobility activities for men, but not for women [13]. Two more recent studies relied on annual assessment data from the Rush Memory and ageing project [7, 14]. Lung function as measured by a combination of forced expiratory volume, forced vital capacity, and peak expiratory flow contributed significantly to the prediction of incident mobility (defined by measured gait speed) over an average of 4 years of follow-up, even in proportional hazard models that also included measures of respiratory and leg muscle strength [7]. In a linear mixed-effects analysis of change in walking ability over an average of 3 years, greater respiratory muscle strength was associated with slower rates of decline in mobility [14].

Taken together, these studies suggest a chain of events from lung function to mobility in late adulthood, although the average longitudinal follow-up period was at most 5 years. Moreover, none of these studies tested alternative models of the causal chain in which changes in motor function contributed to subsequent lung function. The disablement process model includes the possibility of feedback loops from disability to functional limitations and impairments [6], and studies suggest that physical function predicts age-related decline in lung function (e.g., [15]. To our knowledge, however, only one study to date has tested alternate models of the chain of events. Sillanpää and colleagues [16] used cross-sectional data to test three different path models of the relationships among muscle strength, measured mobility, and lung function. Model comparisons indicated that the model including pathways from muscle strength to lung function to mobility provided the best fit to the data.

The goal of the current analysis was to conduct a similar comparison of alternative temporal models of the relationship between lung function and motor function using longitudinal data from the Swedish Adoption/Twin Study of ageing [17]. SATSA includes up to nine waves of testing for a follow-up period of up to 27 years. In addition to standard spirometry measures, SATSA collected 24 different measures of motor function tapping balance, flexibility, and fine motor skills. These data were used to test models incorporating bidirectional longitudinal influences between lung and motor function to test the temporal chain of events in the disablement process.

## Methods

### Participants

Accrual procedures for the Swedish Adoption/Twin Study of ageing (SATSA) have been described previously. In brief, the sample was recruited from the population-based Swedish Twin Registry [17]. In-person testing (IPT) took place in a location convenient to the participants and was completed during a single 4-h visit. At IPT2 through IPT5, additional twins who had reached age 50 since the last wave were invited to participate in SATSA. Intervals between testing waves ranged from 2 to 7 years; the total time span from IPT1 to IPT10 was 27 years (note that IPT4 had a reduced sample and, thus, is not included in the current analyses). A total of 829 participants had both lung and motor functioning data from at least one wave; mean number of waves was 4.87 (SD = 2.63) and mean follow-up was 13.54 years (SD = 8.71). Eighty percent of the sample participated in three or more waves. Sixty percent of the sample was female and there were no sex differences in mean waves of participation [*t *(827) = 0.39, *n.s.*] or mean follow-up [*t*(827) = 1.12, *n.s.*]. Mean ages at each wave are presented in Table 1.

**Table 1:** Sample characteristics at each wave of testing

Wave	*N*	Age Mean (SD)	PEF Mean (SD)	Balance Mean (SD)	Flexibility Mean (SD)	Fine motor Mean (SD)
IPT1	531	64.82 (8.30)	368.19 (106.63)	10.83 (2.17)	2.02 (0.21)	8.65 (1.48)
IPT2	551	65.74 (8.91)	370.49 (108.95)	10.70 (1.97)	2.19 (0.52)	8.55 (1.21)
IPT3	546	68.64 (9.20)	366.32 (126.83)	10.98 (2.14)	2.25 (0.58)	8.67 (1.31)
IPT5^a^	506	70.11 (9.66)	339.97 (139.23)	11.74 (3.75)	2.29 (0.77)	9.02 (2.50)
IPT6	398	71.35 (8.77)	363.75 (146.87)	11.76 (3.30)	2.32 (0.71)	9.31 (2.23)
IPT7	348	73.95 (8.74)	359.09 (131.15)	12.34 (4.21)	2.45 (0.87)	9.81 (3.39)
IPT8	301	75.02 (8.12)	366.14 (130.67)	13.56 (5.53)	2.60 (1.16)	–^b^
IPT9	259	76.40 (7.94)	363.92 (123.22)	14.00 (5.91)	2.69 (1.27)	–^b^
IPT10	242	77.70 (7.56)	359.68 (123.17)	13.59 (5.20)	2.48 (0.87)	–^b^

^a^IPT4 had insufficient data to be included in analyses

^b^Fine motor factor was not collected at waves 8–10

Data were divided into fourteen 3-year age intervals from ages 50 to 89. The data were divided into age intervals, such that everyone with data (regardless of wave) at ages 50–52.9 was included in the first age interval, labeled “50.” Sample sizes within the age intervals are maximized in the middle of the age range (60–80). The data become too sparse after age interval “89” (i.e., after age 91.9) to support statistical modeling; therefore, only data up to age interval “89” were included in these analyses. Sample sizes and means for lung and motor functioning (translated to T scores) in each age interval are provided in Supplemental Table 1.

### Measures

#### Motor functioning

Twenty-four measures of motor functioning were collected at each wave. Data reduction included two components [18]. First, analyses indicated that nurse ratings of successful performance (1 = no difficulty, 2 = some difficulty, and 3 = impossible) were more sensitive to subtle changes with age than performance time [18, 19]. In other words, timed performance of young–old adults on these measures did not vary extensively; in contrast, qualitative ratings demonstrated more variance across the entire age range included in these analyses. Therefore, nurse ratings of performance were used, instead of timing data, to generate the motor factors. Second, factor analyses were compared across wave and age to identify the most consistent and interpretable factor solution, resulting in a three-factor solution incorporating 20 of the items. The remaining four items did not load consistently on any factor. The flexibility factor included two items: touch left earlobe with right hand behind the head, and vice versa (scores can range from 2 to 6). The Fine Motor Movement factor included eight measures of motor functioning: pour water from a jug into a glass, pour water from one hand to the other (both dominant and non-dominant hands), insert key into lock and turn, insert electrical plug into socket, screw in a light bulb, put coins in a coin slot, and dial the numbers 1–9 on a rotary phone (scores can range from 8 to 24). The balance factor included ten measures of motor functioning and can also be considered a measure of gross motor function: walk and turn 3 m, single chair stand, five chair stands, standing balance with feet side-by-side for up to 10 s, standing balance with feet together and arms extended for up to 10 s, lift a glass, lift a 1 kg packet, pick up a pen from the floor from a standing position, touch right fingers to left toes while seated, and vice versa (scores can range from 10 to 30). Mean scores on the motor factors at each wave are presented in Table 1.

For use in the statistical model, factor scores were transformed to T-score metric using mean and standard deviation at wave 1 to ensure a consistent metric; higher scores indicate more difficulties completing the tasks. Only two of the eight measures that make up the Fine Motor factor were collected at waves 8–10; therefore, fine motor factor scores could not be calculated for those waves of participation. The age range available for fine motor does not differ from the other motor factors; however, the coverage of data in the later ages is thinner. As a result, only 12 age intervals were used in modeling longitudinal trajectories for fine motor.

#### Lung functioning

Multiple measures of lung function are available; however, peak expiratory flow (PEF) taps intrinsic pulmonary function, whereas measures of vital capacity measure both lung function and respiratory muscle strength [20]. Lung function was tested on portable spirometers with subjects in seated position and their nasal passages blocked with nose clips. Two trials of PEF were completed and data from the best trial were used in the present analyses. At wave 2, forced expiratory volume in the first second (FEV1) was assessed instead of PEF. As both measures were available from all other waves, these data were used to create a linear transformation equation and FEV1 at wave 2 was translated to PEF units. Mean PEF values (L/min) at each wave are presented in Table 1. An apparent dip in PEF at IPT5 resulted from changes in the sample via both drop-out and the addition of new participants, as well as the longer interval between IPT3 and IPT5. As shown in Supplemental Table 1, when considered across age instead of wave, PEF demonstrates the expected monotonic decrease with age. For use in the statistical model, PEF was corrected for body volume through dividing it by the individual’s squared height in meters [21] and the value was then transformed to T-score metric using mean and standard deviation at wave 1. The purpose of the current analysis was to examine longitudinal covariation between lung and motor function, without over-correcting for possible contributions to mean-level function in either domain. Thus, we chose not to correct either lung or motor function for, say, smoking status, self-rated health, activity levels, etc. Correlations between PEF and the motor factors at each wave are presented in Supplemental Table 2. Across waves, lower scores on PEF were associated with more difficulties on all three motor factors.

### Statistical method

**Fig. 1:**
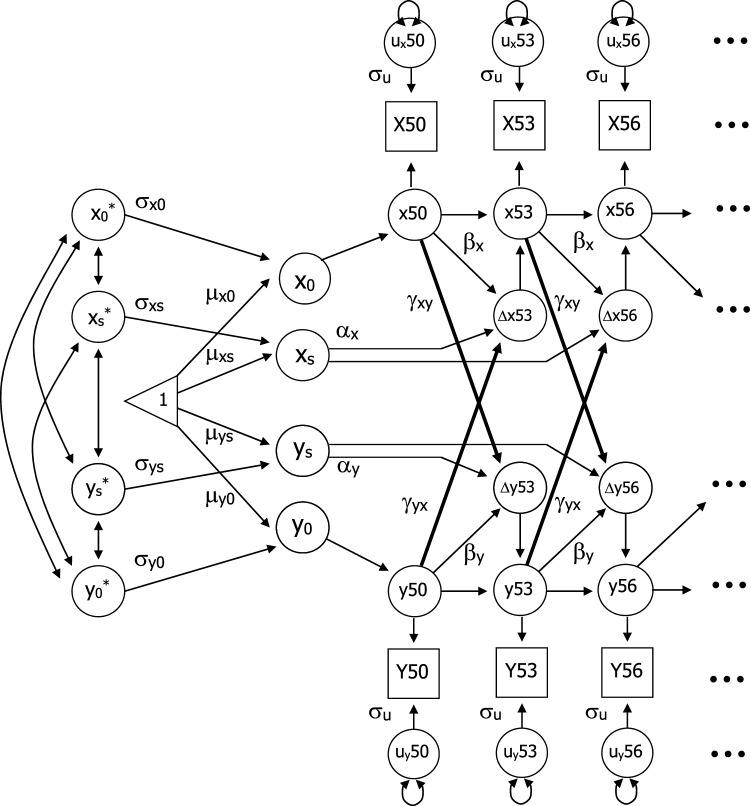
Bivariate dual change score model: *Y*_*0*_ represents the intercept of the trajectory and *Y*_s_ represents the linear slope; *Y*_0_* and *Y*_s_* represent standardized intercept and slope. Mean (*μ*) and standard deviation (*σ*) are estimated for each intercept and slope. *Y*50 represents observed performance on measure *Y* at age 50, with *y*50 indicating the latent true score and *u*_y_50 signifying error. Three-year age segments from *Y*50 through *Y*86 were included in the model. Error variance (*σ*_u_) is assumed to be constant at each age. Change in performance *(∆y*53) is a function of constant change (*α*), proportional change (*β*), and a coupling mechanism (*γ* and bolded paths) where change in trait *X* depends on the previous value of *Y*, and vice versa

Bivariate dual change score models (DCSM) were used to examine the bivariate relationship between lung and motor functioning. Extensive discussions of the model are available [22, 23], as well as comparisons of DCSM with latent growth curve models [24, 25]. As presented in Fig. 1, the model is based on latent difference scores that create a growth curve based on change from one age to another age (∆*y*), which is modeled as a function of both constant change (*α*) that accumulates over time in an additive fashion and proportional change (*β*) based on the previous score. Typically, *α* is set to 1 and the parameter *β* differs from zero to the extent that the longitudinal change is nonlinear. The bivariate DCSM allows for a coupling mechanism (*γ*, see bolded paths in Fig. 1) where change in trait *X* depends on the previous value of *Y*, and vice versa.

With the DCSM, it is possible to evaluate hypotheses about temporal order of changes in variables through restrictions on model parameters, while remaining agnostic as to underlying causes of the temporal relationship. Five alternative models were addressed. First, the relationship between the two variables may be bidirectional, such that *X* affects changes in *Y* and *Y* affects changes in *X* (i.e., both *γ*_yx_ and *γ*_yx_ are nonzero). Second, a model including no dynamic coupling among the variables was tested (*γ*_xy_ = *γ*_yx_ = 0). In subsequent models, the dynamic relationship in one direction only is tested (*γ*_yx_ = 0 or *γ*_xy_ = 0) or the cross-variable dynamic effects are equated (*γ*_xy_ = *γ*_yx_)_._

Bivariate DCSM was fit to the data using Mplus Version 7.4 [26]. Model fit was indicated by the log-likelihood (-2LL) and the root-mean-square error of approximation (RMSEA). Adequate fit of the full model to the data is indicated when the RMSEA is less than or equal to 0.1 and an RMSEA of 0.05 or less indicates “close” fit [27]. Nested models were compared using the difference Chi-square test obtained by taking the difference between the obtained model fits (-2LL). The current analyses focused on individual performance by including a correction for twin pairs in the modeling.

## Results

Model fit statistics for testing the five bivariate DCSM models are presented in Table 2. RMSEA indicated adequate fit of the model to the data for all three motor factors: for balance, RMSEA = 0.068 (95% CI = 0.065–0.071), for flexibility, RMSEA = 0.60 (95% CI = 0.057, 0.063), and for fine motor, RMSEA = 0.060 (95% CI = 0.057–0.064). Comparing the no coupling model (model 2) to the full model (model 1) indicated significant reductions in model fit for the relationship between PEF and balance and between PEF and flexibility. No coupling was indicated for the relationship between PEF and fine motor. Additional models were tested to determine the nature of the bivariate relationship between lung function and the motor factors.

**Table 2:** Model fit statistics (-2LL) for the five comparison models testing the bivariate relationships between measures of motor function and PEF

Models	Parameters	Balance	Flexibility	Fine motor
1: Full model	21	− 25,473	− 28,967	− 22,207
2: No coupling	19	− 25,485**	− 28,974*	− 22,208
3: γ Factor → PEF = 0	20	− 25,473	− 28,967	− 22,207
4: γ PEF → factor = 0	20	− 25,482**	− 28,972*	− 22,208
5: Equate coupling	20	− 25,481**	− 28,972*	− 22,208

^*^Change in model fit vs. the full model is significant at *p* < 0.05

^**^Change in model fit vs. the full model is significant at *p* < 0.01

For the relationship of PEF with both Balance and Flexibility, setting the γ motor → PEF parameter to zero (model 3) had no effect on model fit; whereas, setting the γ PEF → motor parameter to zero (model 4) resulted in a significant loss of fit compared with model 1. Change in fit for model 4 versus model 1 was 9.02, *df* = 1, *p* < 0.01 for the balance factor and 4.71, *df* = 1, *p* < 0.05 for the flexibility factor. For the Fine Motor factor, the change in fit for model 4 versus model 1 was not significant (∆fit = 1.75, *df* = 1, *n.s.*). Equating the coupling parameters provides an additional test of the direction of the relationship; beyond testing whether the parameters are significantly different from zero, it tests whether they are significantly different from each other. Again, this test (model 5 versus model 1) resulted significant changes in model fit for balance and flexibility, but not for the fine motor factor. Taken together, these results indicate that lung function precedes and contributes to subsequent changes in the balance and flexibility factors.

Parameter estimates and standard errors from the full bivariate model (model 1) are presented in Table 3. For each bivariate relationship, the estimate of the influence of PEF on the motor factor (γ PEF → factor) is larger than the estimate of the influence of the motor factor on PEF (γ factor → PEF). Although it did not achieve significance for the fine motor factor, this difference between the coupling parameters was in the same direction as the other motor factors, such that γ PEF → Fine Motor was larger than γ fine motor → PEF. The longitudinal relationships between PEF and the motor factors are demonstrated in Fig. 1. Examining the difference in ageing trajectories for the full model versus the model without coupling parameters allows a visual representation of the impact of coupling. The decline trajectory for PEF is nearly identical both with and without coupling in all three bivariate relationships with the motor factors, illustrating the lack of impact the motor factors which have on subsequent age changes in lung function. In contrast, the differences between the ageing trajectories estimated by the full model and the no coupling model are evident for all three motor factors. In each case, when the impact of declining lung function is taken into account in the full coupling model, the increase in difficulties in motor function with age is reduced. The same pattern is evident even for the fine motor factor, although the lack of data at waves eight through ten resulted in truncated trajectories that did not achieve statistical significance. Thus, the bivariate DCSM indicates that lung function influences subsequent age changes in motor function, such that reduced lung capacity contributes to more difficulties in motor function at subsequent testing waves.

**Table 3:** Parameter estimates (SE) from the bivariate dual change score model between PEF and the motor factors (model 1)

Parameters	PEF (with Balance)	Balance	PEF (with Flexibility)	Flexibility	PEF (with Fine motor)	Fine motor
Mean intercept, μ_0_	56.14 (0.57)	49.37 (0.62)	56.47 (0.54)	53.72 (1.35)	56.06 (0.54)	47.27 (0.50)
Mean slope, μ_s_	− 2.60 (1.66)	− 1.50 (4.61)	− 2.03 (1.31)	5.22 (6.68)	0.02 (4.44)	− 35.42 (8.33)
Proportional change, β	0.03 (0.02)	0.36 (0.03)	0.02 (0.02)	0.26 (0.03)	0.03 (0.04)	0.58 (0.09)
Coupling, γ PEF → factor		− 0.30 (0.07)		− 0.35 (0.11)		0.15 (0.08)
Coupling, γ factor → PEF	− 0.00 (0.01)		− 0.00 (0.01)		− 0.05 (0.06)	
Error deviation, σ_u_	5.71 (1.03)	9.29 (1.64)	5.68 (1.02)	23.46 (3.96)	5.56 (1.02)	9.09 (1.74)

## Discussion

To systematically test the chain of events predicted by the disablement process, dual change score models were used to test the temporal relationships between lung function and motor function in longitudinal data. Comparison of models incorporating influences from lung function to subsequent motor function and from motor function to subsequent lung function indicated clearly that the chain of events moved in one direction: reduction in lung function contributes to subsequent increases in difficulties in motor function. Possible mechanisms for this relationship include systemic changes that affect both lung function and motor function [16] or more specific influences of declining lung function on the vigor required to complete motor function tasks [7, 8].

It is important to consider the connection between lung function and motor function as one link in a larger chain of events. A wider-angle interdisciplinary lens, incorporating both physical and cognitive components, is required to fully understand the trajectory of functional consequences that over time lead from independence to dependence [6, 28]. Studies have found relationships among personality, cognition, lung function, and motor function in various configurations of predictor and outcome in both cross-sectional and longitudinal analyses [3, 10, 12]. Piecing together, the individual links in the chain from multiple perspectives can provide a possible model for the cascade of events that tend to result in disablement and dependence. For example, a recent meta-analysis examining the results of 40 longitudinal studies of the relationship between physical and cognitive functioning found stronger evidence that baseline physical function predicted changes in cognitive function than the reverse [29]. Similarly, a coordinated analysis of eight longitudinal studies of lung function and cognitive function found a consistent link between changes in the two domains [30]. In addition, the previous applications of the dual change score model to SATSA data have indicated separate unidirectional steps in a possible chain events: processing speed contributed to subsequent changes in cognitive function [31], lung function contributed to subsequent changes in processing speed [32], and motor function contributed to subsequent changes in processing speed [33]. The current analysis adds to a growing body of literature indicating that lung function contributes to subsequent motor function and mobility. Taken together, these studies suggest a possible cascade of events: declining lung function results in increasing difficulties in motor function, which contribute to subsequent declines in processing speed, which underlie changes associated with cognitive ageing. The end result, arising from both physical and cognitive changes, will be reduced independence and greater reliance on assistance in some form.

**Fig. 2:**
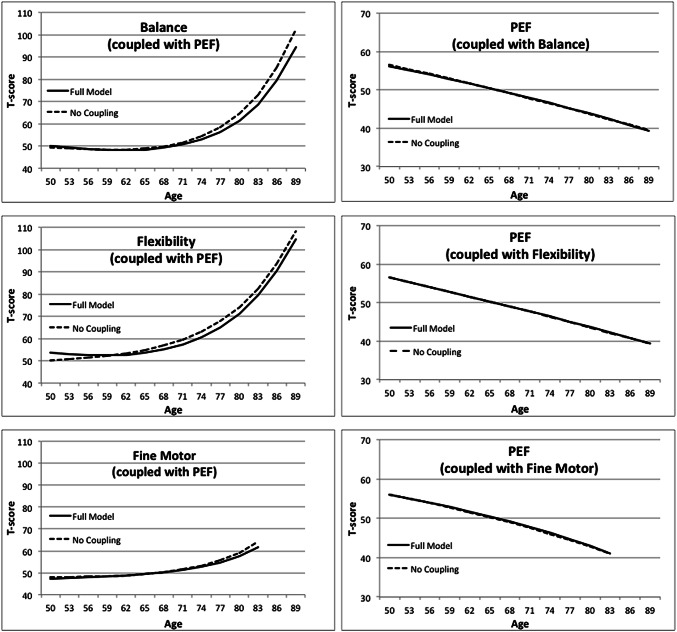
Change trajectories estimated for the motor factors and PEF by the model, with and without coupling

Although DCSM is considered to have many important advantages over other methods for addressing hypotheses about dynamic relationships among variables [34], the method is also limited by many of the statistical assumptions common to structural equation models. The data are assumed to be missing at random, the sample is assumed to be relatively homogeneous, and structural relations based on interindividual variance and on intraindividual variance are assumed to be equivalent [25]. Differences between the two major variables in reliability, change with age, or measurement level could impact the fit of the bivariate DCSM. Particularly relevant here is the difference in measurement level between lung function and motor function. PEF measures an individual’s maximal effort, which may be more sensitive to changes with age. The motor factor measures that had a maximal time limit (e.g., balance for 10 s) by definition did not measure an individual’s maximal effort. Only 2 of the 20 measures of motor functioning would be subject to this limitation; however, nurse ratings of performance will not have the same level of rigor and reliability that a physiological measure like PEF has. Nurse ratings were used instead of time to complete the tasks, because they demonstrated more sensitivity to performance difficulties across the range of ages included in SATSA than the timing measures. Moreover, as demonstrated in Fig. 2, the motor factors demonstrated increases in difficulty with age greater in magnitude than the age changes shown by PEF. As a result, conclusions from the bivariate DCSM should be interpreted with some caution.

As with any longitudinal sample, attrition occurred in SATSA. Even though the sample was representative of the population at intake, non-random drop-out through the course of the longitudinal studies results in increasingly select samples of adults who are healthy enough to participate. In SATSA, research nurses visited the participants at their current residence; therefore, data collection could continue even after onset of illness or entry in to care. As a result, wave-to-wave drop-out was quite low (about 8%), but drop-out accumulates across waves. Consequently, our analyses have likely underestimated the extent of change with age.

The use of qualitative assessment of motor functioning instead of timed assessment could be considered both a strength and a weakness of the current analyses. Qualitative assessment of performance by trained nurses produced more nuanced assessment of physical performance: for example, a stopwatch does not capture the “wobbles” that an observer can see during a balance task. Similarly, reports of fatigue or increased time required to complete activities of daily living can be more informative than simple success or failure [8]. The qualitative assessment used here resulted in sufficient variability to support factor analysis [18] and structural modeling across the entire age range included in the current analyses. However, regardless of how well trained the nurse-interviewers were and how little staff turnover occurred during SATSA (12 interviewers over 27 years), observer ratings are unlikely to be as reliable as timed measures of performance.

Combined with previous results, these results suggest a pathway that may start with age declines in lung function, impact motor function, and then processing speed, finally resulting in cognitive ageing. These results indicate that assessments of older persons, such as the Comprehensive Geriatric Assessments [35], should include lung function to identify individuals at risk at an earlier stage. Moreover, if reduced pulmonary function is identified, it should lead to a more detailed evaluation of motor function and a search for factors that may influence both lung and motor function. In turn, interventions focusing on improving or maintain lung function should have the added effect of maintaining motor and cognitive function and delaying the onset of dependence and need for care. Whereas in previous centuries, only the most vigorous (and luckiest) individuals survived to late old age, health advances of the twentieth century have allowed frailer and more vulnerable individuals to live longer [2, 36]. Appropriate treatment and strategic interventions can ensure that longer life does not necessarily mean a longer period of morbidity and decline [1, 6]. Our results suggest that interventions focused on lung function could have positive impacts on functioning in multiple domains. Understanding the cascade of events that can lead to dependence can help in the development of interventions targeted early in the disablement process.

## Electronic supplementary material

Below is the link to the electronic supplementary material.

Supplementary file1 (DOCX 17 kb)
