# Supplementary material for: Role of motor function and lung function in pathways to ageing and decline
Source: Aging Clin Exp Res. 2020 Feb 13;32(12):2479–87. doi: 10.1007/s40520-020-01494-3 (PMC7680325; doi:10.1007/s40520-020-01494-3)
Supplement: Supplementary file 1 — Supplementary file1 (DOCX 17 kb) [file 40520_2020_1494_MOESM1_ESM.docx]

Supplemental Material for

Role of motor function and lung function in pathways to aging and decline

Deborah Finkel, Marie Ernsth-Bravell, and Nancy L. Pedersen

Table 1: Descriptive statistics at each age interval for PEF and the motor factors.

Table 2: Correlations between PEF and the motor factors at each wave.

Supplemental Table 1. Descriptive statistics at each age interval for PEF and the motor factors.

| Interval | N | Mean PEF (SD) | Mean Balance (SD) | Mean Flexibility (SD) | Mean Fine Motor (SD) | N Fine Motor |
| --- | --- | --- | --- | --- | --- | --- |
| 50 | 92 | 56.26 (10.90) | 47.92 (10.03) | 52.75 (22.19) | 47.99 (5.41) | 92 |
| 53 | 158 | 55.64 (9.08) | 47.28 (4.18) | 51.95 (14.69) | 47.01 (4.48) | 158 |
| 56 | 195 | 55.47 (9.61) | 48.56 (8.54) | 53.25 (19.05) | 47.70 (8.42 | 195 |
| 59 | 236 | 54.97 (10.03) | 48.88 (6.97) | 53.62 (17.61) | 48.27 (8.50) | 236 |
| 62 | 320 | 52.58 (10.80) | 48.49 (6.29) | 53.33 (17.59) | 48.76 (8.26) | 310 |
| 65 | 397 | 51.37 (11.05) | 49.64 (8.89) | 55.47 (19.89) | 50.02 (11.63) | 371 |
| 68 | 394 | 50.71 (21.16) | 50.16 (10.04) | 57.52 (21.90) | 51.21 (12.15) | 364 |
| 71 | 397 | 48.11 (11.46) | 52.02 (11.62) | 59.11 (27.55) | 50.38 (7.21) | 335 |
| 74 | 369 | 46.44 (11.59) | 53.96 (14.82) | 63.58 (33.22) | 52.57 (11.41) | 304 |
| 77 | 274 | 45.92 (11.48) | 57.87 (18.39) | 68.84 (36.42) | 53.97 (13.82) | 238 |
| 80 | 213 | 44.73 (11.77) | 61.35 (20.47) | 73.51 (46.51) | 57.47 (17.13) | 190 |
| 83 | 158 | 43.67 (11.93) | 66.04 (25.09) | 83.48 (55.93) | 60.04 (18.26) | 116 |
| 86 | 103 | 41.12 (12.92) | 75.07 (29.13) | 93.45 (60.69) | --^a^ |  |
| 89 | 40 | 37.76 (11.49) | 87.12 (33.14) | 110.02 (71.80) | --^a^ |  |

Note: Age interval “50” includes ages 50 – 52.9 ^a^ Only 12 age intervals were used for the Fine Motor factor

Supplemental Table 2. Correlations between PEF and the motor factors at each wave.

| Wave | N | PEF x Balance | PEF x Flexibility | PEF x Fine Motor |
| --- | --- | --- | --- | --- |
| IPT1 | 531 | -.15** | -.07 | -.25** |
| IPT2 | 551 | -.23** | -.13** | -.15** |
| IPT3 | 546 | -.21** | -.20** | -.25** |
| IPT5^a^ | 506 | -.17** | -.10* | -.17** |
| IPT6 | 398 | -.23** | -.11* | -.21** |
| IPT7 | 348 | -.39** | -.26** | -.22** |
| IPT8 | 301 | -.25** | -.21** | --^b^ |
| IPT9 | 259 | -.27** | -.19** | --^b^ |
| IPT10 | 242 | -.24** | -.14* | --^b^ |

^a^ IPT4 had insufficient data to be included in analyses

^b^ Fine Motor factor was not collected at waves 8 through 10

* *p* < .05

** *p* < .01
